# Relationship of Blood Pressure With Mortality and Cardiovascular Events Among Hypertensive Patients aged ≥60 years in Rural Areas of China: A Strobe-Compliant Study

**DOI:** 10.1097/MD.0000000000001551

**Published:** 2015-10-02

**Authors:** Liqiang Zheng, Jue Li, Zhaoqing Sun, Xingang Zhang, Dayi Hu, Yingxian Sun

**Affiliations:** From the Department of Clinical Epidemiology, Library, Shengjing Hospital of China Medical University, Shenyang (LZ); Department of Epidemiology, Tongji University Medical School, Shanghai (JL, DH); Department of Cardiology, Shengjing Hospital of China Medical University (ZS, YS); and Department of Cardiology, the First Affiliated Hospital of China Medical University, Shenyang, P.R. China (XZ).

## Abstract

The Eighth Joint National Committee (JNC-8) panel recently recommended a systolic blood pressure (BP) threshold of ≥150 mmHg for the initiation of drug therapy and a therapeutic target of <150/90 mmHg in patients ≥60 years of age. However, results from some post-hoc analysis of randomized controlled trials and observational studies did not support these recommendations.

In the prospective cohort study, 5006 eligible hypertensive patients aged ≥60 years from rural areas of China were enrolled for the present analysis.

The association between the average follow-up BP and outcomes (all-cause and cardiovascular death, incident coronary heart disease [CHD], and stroke), followed by a median of 4.8 years, were evaluated using Cox proportional hazards models adjusting for other potential confounders. The relationship between BP (systolic or diastolic) showed an increased or J-shaped curve association with adverse outcomes. Compared with the reference group of BP <140/90 mmHg, the risk of all-cause death (hazard ratio [HR]: 2.698; 95% confidence interval [CI]: 1.989–3.659), cardiovascular death (HR: 2.702; 95% CI: 1.855–3.935), incident CHD (HR: 3.263; 95% CI: 2.063–5.161), and stroke (HR: 2.334; 95% CI: 1.559–3.945) was still significantly increased in the group with BP of 140–149/<90 mmHg.

Older hypertensive patients with BP of 140–149/<90 mmHg were at higher risk of developing adverse outcomes, implying that lenient BP control of 140–149/<90 mmHg, based on the JNC-8 guidelines, may not be appropriate for hypertensive patients aged ≥60 years in rural areas of China.

## INTRODUCTION

Hypertension is an important modifiable risk factor for cardiovascular disease (CVD) and all-cause mortality in the Chinese population.^1^ During recent decades, a number of randomized controlled trials (RCTs) have supported the benefits of antihypertensive medication on reducing the incidence of CVD among the elderly.^2–5^

Recently, the Eighth Joint National Committee (JNC-8) panel members suggested that hypertensive patients ≥60 years of age with a systolic blood pressure (SBP) of ≥150 mmHg should initiate the drug therapy and the optimal blood pressure (BP) goal should be of <150/90 mmHg. The recommendations claim that “setting a goal SBP of <140 mmHg in this age group provides no additional benefit compared with a goal SBP of 140 to 160 mmHg or 140 to 149 mmHg.”^6^ However, some JNC-8 panel members (5 of 17) disagreed with this standpoint.^7^ In addition, the 2013 European Society of Hypertension/European Society of Cardiology guidelines for the management of hypertension^8^ also recommended that the therapeutic target of SBP should be of 140 to 150 mmHg in elderly hypertensive aged ≥80 years with SBP ≥160 mmHg.^8^ Experts have also put emphasis on the comment that the data making definitive recommendations on targeted BP levels in the elderly is very limited.^9–12^ Furthermore, results from some post-hoc analysis of RCTs and observational studies did not support the recommendations.^13–15^ The RCTs to provide definitive evidence on the optimal BP target for elderly hypertensive patients are few and the optimal BP goal among the elderly remains controversial.^2,3,16,17^

Furthermore, the majority of evidence used as the basis for the management of hypertension is obtained from RCTs, and these results are limited in their application to the general patients due to the strict inclusion and exclusion criteria. The “real-world” effects from RCTs should be validated using large-scale observational epidemiological studies. Therefore, we selected a representative cohort hypertensive sample aged ≥60 years in rural areas of China to investigate the relationship between BP and adverse outcomes.

## METHODS

### Study Population and Study Design

This is a large-scale epidemiological prospective study. A multistage, random cluster sampling design was performed to select a representative sample of the rural population aged ≥35 years from 84 rural villages in Liaoning Province from 2004 to 2006. The detailed methodology was described elsewhere.^18^ In 2008 and 2010, all subjects were invited to participate in the biennial follow-up study. Of the 45,925 subjects at baseline, 3579 were not included in the follow-up study due to lack of contact information. Overall, 42,346 aged ≥35 years at baseline examination were deemed eligible to participate in the follow-up study. From this population, a total of 39,845 (94.1%) study participants (or their guardians) were identified and agreed to be interviewed as a part of the follow-up study. The 6080 subjects who were lost to follow-up relative to the participants who have completed the follow-up study (n = 39,845) have the older age (median: 52.0 versus 50.2 years; *P* < 0.001), higher SBP (median: 134 versus 131 mmHg; *P* < 0.001) and diastolic BP (DBP) (median: 85 versus 82 mmHg; *P* < 0.001), and were more likely to be female (53.0% versus 50.1%; *P* < 0.001). For the present study, no-hypertensive subjects (n = 24,304) and baseline age <60 years (n = 30,760) at baseline were excluded, leaving 5066 data from hypertensive patients aged ≥60 years at baseline for the present analysis.

### Ethical Approval, Informed Consent, and Patient Privacy

The research protocol was approved by China Medical University Research Ethics Committee and written informed consent was formally obtained from all subjects or their guardians. We explained the contents of informed consent to subjects who agreed to participate the present study which included the purpose of the study, confidentiality agreement of personal information, and medical items. We did not include the personal information and patient's identity with the other data. Only the principal investigator had access to this information. No reference to the patient's identity was made at any stage during data analysis or in the paper.

### Data Collection and Physical Examinations at Baseline

At baseline examination, all participants were recruited and examined at a single clinic visit by their local doctors in their geographical area of origin from 2004 to 2006. There was a central steering committee in place to run the study, with an independent subcommittee for quality control. With the help of health bureau of local governments, we recruited strictly the local doctors who were willing to become investigators and were physicians who had at least 3 years of experience. Before the survey was performed, all the eligible doctors were invited to attend an organized training and certification process in Fuxin County. Our research staff trained the local doctors by way of lecture, role playing, and simulation training. The training content included the purpose of this study, how to administer the questionnaire, standard methods of measurement, the importance of standardization, and study procedures. A strict test which composed of written examination and standard operation evaluation was conducted after the training. Only those who scored perfectly (the simulation test error rate = number of mistaken items/number of total items <2%) on the test and mastered soundly the main points of standard operations such as BP measurements and physical examinations were recruited become investigators. A total of 98 local eligible doctors were invited to participate in the research, and 96 of them passed the test. For 24 doctors came from the same village (2 doctors per 1 village), those who scored higher was the final investigator in the village. Finally, a total of 84 local doctors from 84 rural villages (one doctor per one village) who received strict training and passed the assessment became the investigators. During data collection, the research staff continued monitoring the doctors and their performance, even after the considerable initial training.

Data on demographic variables (age, sex, and ethnicity), smoking status, use of alcohol, information on duration of hypertension, antihypertensive medications, lipid-lowering drug use, and history of stroke and coronary heart disease (CHD) at baseline were obtained by interviews with a standard epidemiological questionnaire. Drinking status was assessed by alcohol consumption which defined as the weekly consumption of beer, wine, and hard liquor converted into grams of alcohol. Alcohol drinker was defined as alcohol consumption at least 8 g per week during the last year. Current smoking was defined as people who smoked at least 1 cigarette every day and continued for at least 1 year. Smoking was assessed as a part of the questionnaire. The individuals were asked whether they currently smoked or not (Do you smoke currently?). Body weight and height were measured with subjects wearing light clothing and without shoes according to a standard protocol. History of stroke or CHD at baseline was positive if a stroke or CHD was reported during the baseline interview and confirmed by medical records. Body mass index (BMI) was calculated by the weight in kilograms divided by height in square meters. Diabetes and hypercholesterolemia were defined according to self-report or medical records.

### BP Measurements at Baseline

BP was measured using a standardized automatic electronic sphygmomanometer (HEM-741C; Omron, Tokyo, Japan) and 1 of 4 cuff sizes (pediatric, regular adult, large, or thigh) chosen based on the basis of arm circumference. A trained and certified observer used an American Heart Association protocol to perform 3 BP measurements after study participants had been seated quietly for 5 minutes. Participants were instructed to avoid alcohol consumption, cigarette smoking, coffee/tea, and exercise for at least 30 minutes before these measurements. The mean of 3 BP values was calculated and used for all subsequent analysis. The terminal digit preference was used as an indicator of quality control for BP measurements in the present study. Participants with hypertension at baseline were defined as they had an average SBP ≥140 mmHg, and/or an average DBP ≥90 mmHg, and/or use of antihypertensive medications within the previous 2 weeks.

### Follow-Up

All study subjects were invited to attend the biennially follow-up study: from January to June 2008; and from July to October 2010. At each visit, we collected the information on clinical end points, adverse events, and concurrent medication use. During each visit, BP measurements were taken and recorded according to a standard protocol identical to that of the baseline examination. For the present analysis, we used all postbaseline BP measurements to calculate the average follow-up SBP and DBP for each patient. The baseline value was substituted for patients without postbaseline BP data. We then evaluated the risk of study outcomes according to BP.

### Study Outcomes

The present study outcomes included all-cause mortality, CVD mortality, incident stroke, and CHD. Deaths were identified through hospital records and by directly contact with their families. Using the International Classification of Diseases, 9th Revision, Clinical Modification, deaths due to CVD were assigned a code from 400 through 444 including CHD, stroke, and others. We confirmed that death from CVD on the basis of autopsy reports, death certificates, medical record abstract, or information obtained from family members.

Stroke was defined according to the WHO Multinational Monitoring of Trends and Determinants in Cardiovascular Disease criteria: a sudden onset of focal (or global) disturbance of cerebral function lasting >24 hours (unless interrupted by surgery or death) with no apparent nonvascular cause. The definition included patients presenting with clinical signs and symptoms suggestive of complete stroke, including ischemic stroke and hemorrhage stroke. Transient ischemic attacks and silent brain infarctions (cases without clinical symptoms or signs) were not included; neither were events associated with trauma, blood disease, or malignancy. Most stroke cases (n = 479, 97.6%) were diagnosed by computed tomography, magnetic resonance imaging (including diffusion image), magnetic resonance angiography of the brain, and carotid duplex imaging. CHD was defined as myocardial infarction, angina pectoris of which patients received treatment in hospital for ischemic discomfort and diagnose by coronary angiography, coronary revascularization, and sudden death. Coronary revascularization was achieved when a patient underwent percutaneous coronary intervention (eg, angioplasty, stenting, atherectomy, and laser ablation) or coronary artery bypass graft. The information was obtained from direct reference to medical records by a single investigator.

All materials were independently reviewed by the end-point assessment committee which included the certified neurologists, cardiologists, and others. All members of the end-point assessment committee were blinded to the study participants’ baseline risk factor information.

### Statistical Analysis

Continuous variables were presented as means and standard deviation, while categorical variables were expressed as percentages. BP values were categorized (SBP, 10 mmHg increments; and DBP, 5 mmHg increments) to study the association between BP and adverse outcomes. Patients groups were compared by χ^2^ tests for categorical variables or one-way analysis of variance for continuous variables, which followed by the least significant difference test for subgroup comparisons. The rates of events were presented as the number of events per 1000 patient-years, based on the ratio of the number of events observed to the total number of patient-years of exposure up to the terminating event or censor.

The decision to use the average BP during follow-up was based on the following: we created 2 separate adjusted models using baseline BP and average follow-up BP. We used Harrell concordance index (C index) to calculate the predictive value for each model and compared them using bootstrapping.^19^ Because the predictive value of models with average follow-up BP (eg, all-cause mortality, 0.702 for SBP and 0.589 for DBP) was significantly better than that of baseline BP (eg, all-cause mortality, 0.564 for SBP and 0.528 for DBP) models, all subsequent mentioning of BP relates to average follow-up BP and not to baseline BP.

We used multivariable Cox proportional hazards models to calculate hazard ratio (HR) with 95% confidence intervals (CIs) for the associations between BP categories and incident events, with the BP category in which the actual event rate was lowest as the reference group. The SBP and DBP were entered the multivariable models separately. We constructed 2 models: in model 1, we adjusted for baseline age and sex; and in model 2, we adjusted for sex, baseline age, Mongolian ethnicity, body mass index, smoking, drinking, antihypertensive treatment, prior CHD, prior stroke, and self-reported history of diabetes and hypercholesterolemia. Next, we divided patients into 3 groups based on BP (≥150/90 mmHg, 140–149/<90 mmHg, and <140/90 mmHg) and repeated the multivariable models, with the <140/90 mmHg category as the reference group. The proportionality assumption was evaluated by scaled Schoenfeld residuals, and the global fit of the models was evaluated by graphically examining the cumulative hazards function relative to the Cox-Snell residuals. All analyses were performed using SPSS statistical software version 13.0 (SPSS Inc., Chicago, IL). A *P* value less than 0.05 was accepted as indicating statistical significance.

## RESULTS

The average baseline age of the study hypertensive patients was 69.5 years (range: 60–95 years) and there were 2571 (51.4%) women. The mean (standard deviation) of baseline DBP and SBP were 91.7 (12.9) mmHg and 162.5 (21.3) mmHg, respectively. At baseline, 28.8% of study hypertensive patients were receiving antihypertensive medications. Other baseline demographics as well as con-concomitant medications were indicated in Tables 1 and 2. Tables 1 and 2 also show baseline characteristics of study patients categorized by average SBP and DBP ranges during follow-up, respectively. Patients with lower mean SBP were more likely to be younger, male, and to have a lower proportion of prior history of stroke (all *P* < 0.05). Patients with lower mean DBP were leaner, and to have a lower proportion of history of CHD (all *P* < 0.05).

**TABLE 1:**
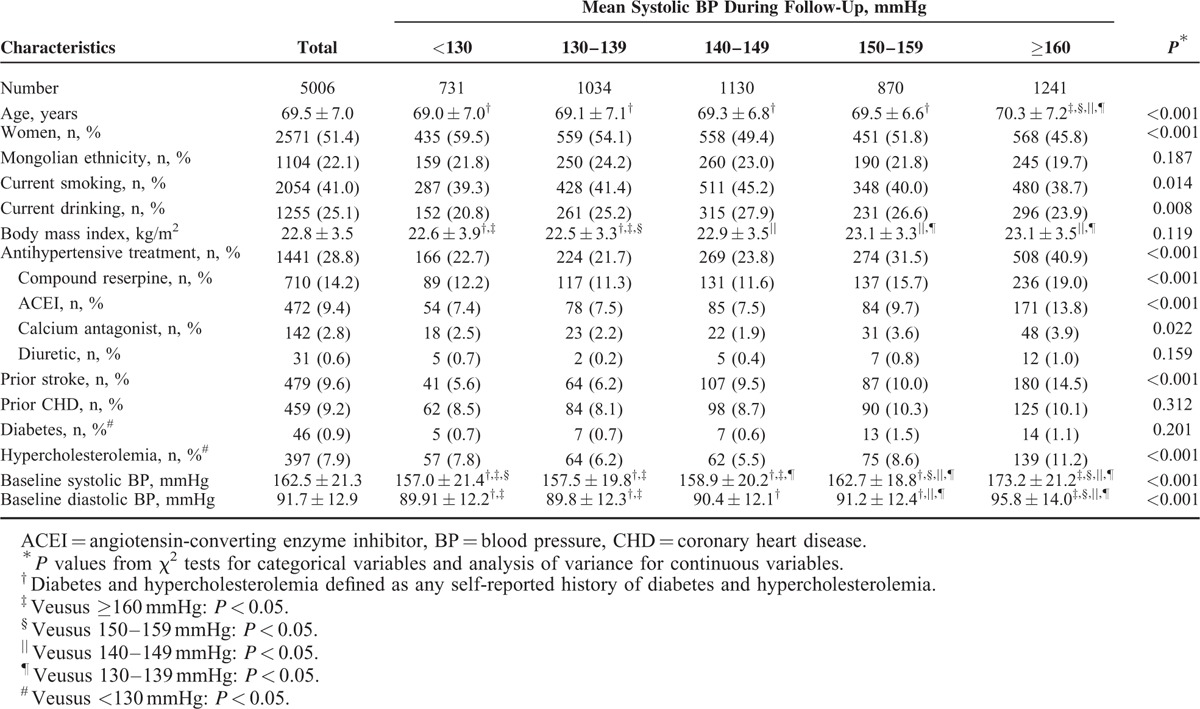
Baseline Characteristics and Demographic of 5006 Study Patients According to Mean Systolic BP Categories

**TABLE 2:**
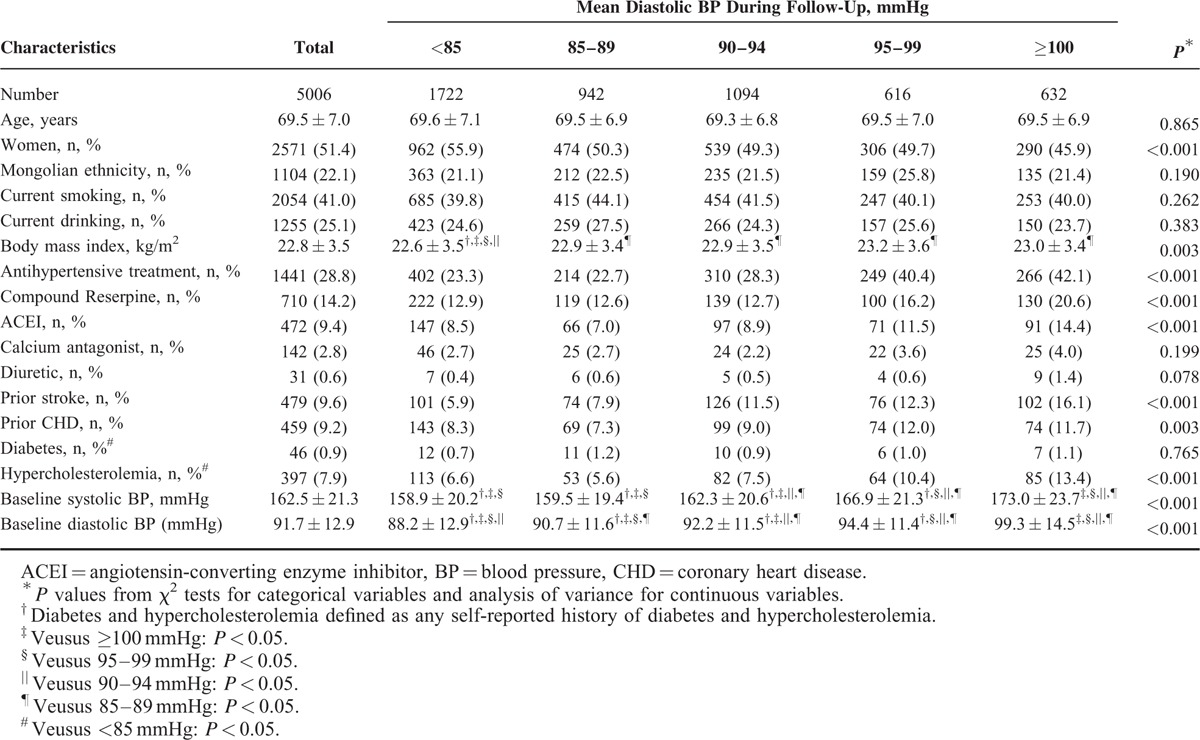
Baseline Characteristics and Demographic of 5006 Study Patients According to Mean Diastolic BP Categories

After a median 4.8 years (range: 1.2–6.5 years) of follow-up and 20,916 person-years of observation, there were 776 deaths and 536 of them attributed to CVD. Mortality from all-cause and CVD were 37.10 (95% CI, 34.54–39.66) and 25.63 (95% CI, 23.48–27.77) per 1000 patient-years, respectively. Meanwhile, 491 incident stroke and 278 incident CHD cases occurred during the follow-up period and the incident rates of stroke and CHD were 23.47 (95% CI, 21.42–25.53) and 13.29 (95% CI, 11.74–14.84) per 1000 patient-years, respectively.

Table 3 shows the rates and HRs (95% CI) of all-cause mortality, CVD mortality, incident CHD, and stroke according to SBP categories. As expected, there was direct correlation between average SBP and incident events. After adjusted by sex and baseline age, patients with SBP of 160 mmHg versus SBP <130 mmHg had HRs of 4.922 (95% CI, 3.662–6.615) for all-cause mortality, 5.143 (95% CI, 3.596–7.356) for CVD mortality, 4.637 (95% CI, 2.848–7.549) for incident CHD, and 5.820 (95% CI, 3.876–8.740) for incident stroke, respectively. In addition, compared with the reference group (SBP <130 mmHg) after adjusted sex and baseline age, the risk of all-cause mortality, CVD mortality, incident CHD, and stroke increased 1.781-fold, 1.767-fold, 2.395-fold, and 2.232-fold in the group with SBP of 140–149 mmHg, respectively. Further adjustment for other baseline potential confounders hardly attenuated the HRs.

**TABLE 3:**
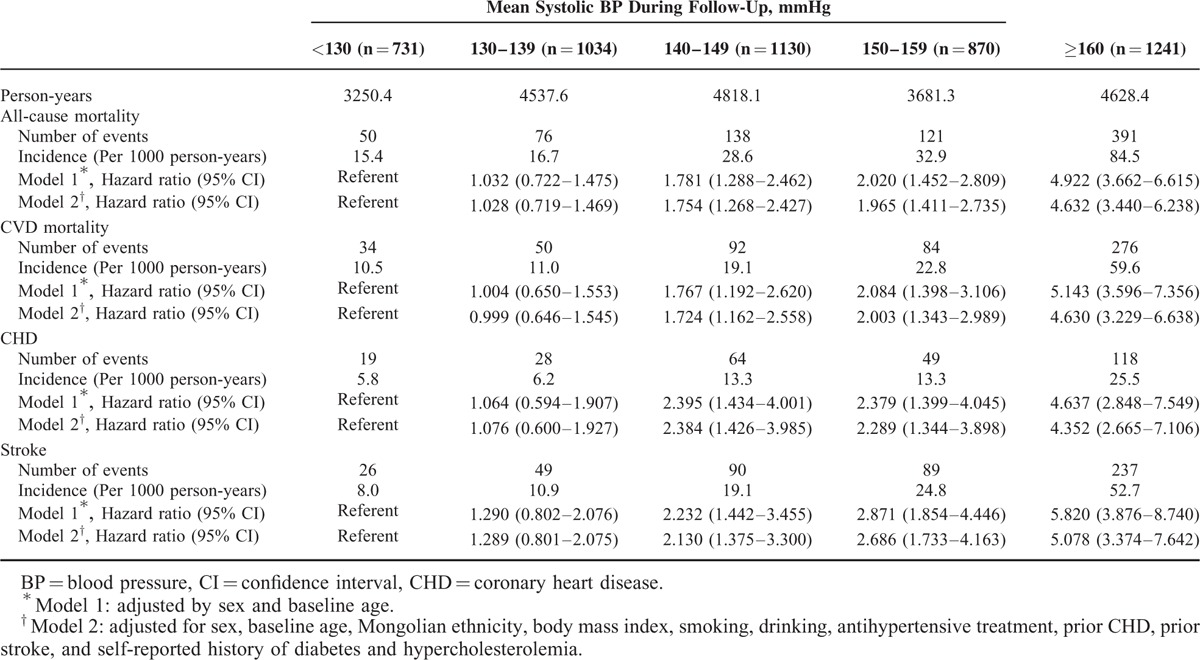
Mean Systolic BP During Follow-Up and Adverse Outcomes

**FIGURE 1:**
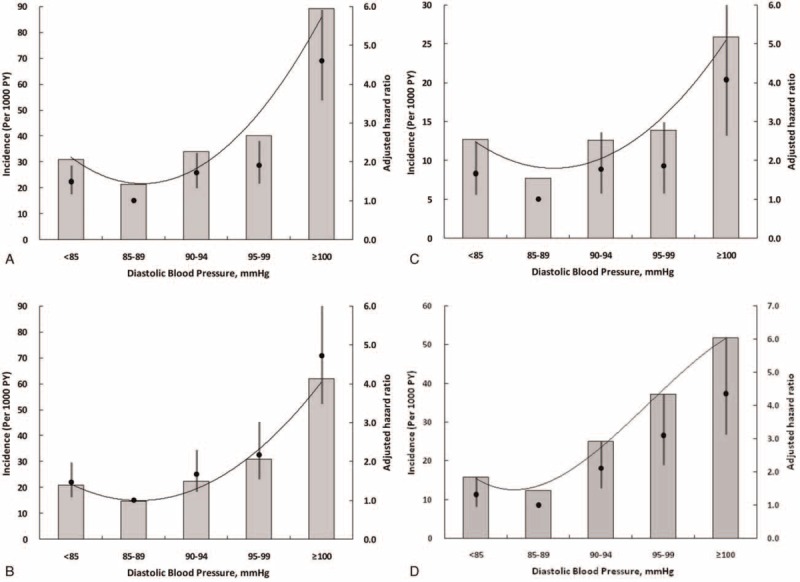
(A) Incidence and adjusted risk of all-cause death as a function of average follow-up diastolic blood pressure. (B) Incidence and adjusted risk of CVD death as a function of average follow-up diastolic blood pressure. (C) Incidence and adjusted risk of coronary, as a function of average follow-up diastolic blood pressure. (D) Incidence and adjusted risk of stroke as a function of average follow-up diastolic blood pressure.

The association between DBP and the incidence and risk of study outcomes followed a J-shaped curve, with an increased event rate (all-cause and CVD mortality, incident CHD) at low and high DBP (Table 4 and Figure 1) compared with the reference group (BP >85–89 mmHg) after adjustment for sex and baseline age, the risk of all-cause mortality, CVD mortality, and incident CHD increased 1.490-fold, 1.469-fold, and 1.665-fold in the group with DBP <85 mmHg and by 4.603-fold, 4.723-fold, and 4.082-fold in the group with DBP >100 mmHg, respectively. Compared with the reference group (BP >85–89 mmHg), after adjusted by sex and baseline age, patients with DBP of 100 mmHg, 95–99 mmHg, 90–94 mmHg, and <85 mmHg had HRs of 4.357 (95% CI, 3.133–6.059), 3.099 (95% CI, 2.205–4.356), 2.108 (95% CI, 1.515–2.933), and 1.322 (95% CI, 0.950–1.839) for incident stroke, respectively. Further adjustment for other baseline potential confounders only changed the HRs slightly.

**TABLE 4:**
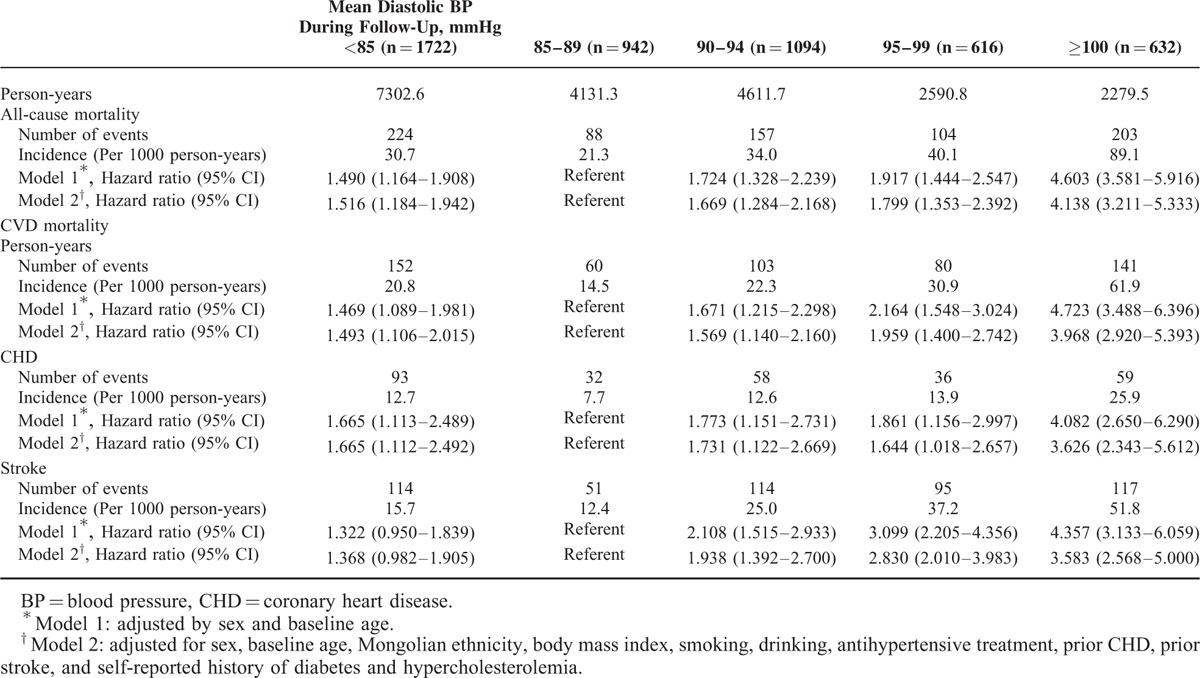
Mean Diastolic BP During Follow-Up and Adverse Outcomes

Next, we evaluated the association between joint distribution of BP (systolic and diastolic) and study outcomes. In the multivariable models adjusted by sex, baseline age and other potential confounders, compared with the reference group of BP < 140/90 mmHg, the risk of all-cause mortality (HR: 2.698; 95% CI: 1.989–3.659), CVD mortality (HR: 2.702; 95% CI: 1.855–3.935), incident CHD (HR: 3.263; 95% CI: 2.063–5.161), and stroke (HR: 2.334; 95% CI: 1.559–3.945) was increased in the group with BP of 140–149/<90 mmHg. Compared with the reference group (BP < 140/90 mmHg), the risk of all-cause mortality, CVD mortality, incident CHD, and stroke increased 3.882-fold (95% CI: 3.077–4.897), 4.026-fold (95% CI: 3.027–5.355), 3.439-fold (95% CI: 2.384–4.960), and 3.836-fold (95% CI: 2.859–5.147) in the group with BP of >150/90 mmHg, respectively.

Table 5 shows the HRs of other associated factors for adverse study outcomes. In the multivariable-adjusted model, women were observed to have the HRs of 0.608 (95% CI, 0.519–0.712) for all-cause mortality, 0.607 (95% CI, 0.503–0.733) for CVD mortality, and 0.520 (95% CI, 0.424–0.637) for stroke, respectively. Increasing age was also noted to be significantly associated with all-cause mortality (HR, 1.619), CVD mortality (HR, 1.698), incident CHD (HR, 1.593), and stroke (HR, 1.352), respectively. Mongolian ethnicity was also associated with a higher risk of all-cause and CVD mortality (*P* < 0.05). No violations of the Cox proportional hazards assumption were observed.

**TABLE 5:**
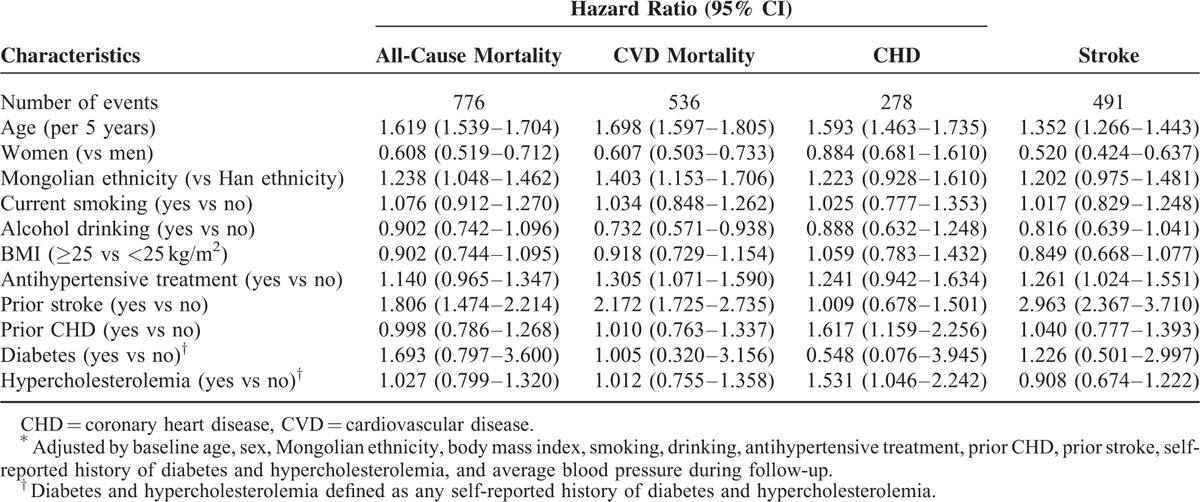
Other Associated Factors With Future Adverse Outcomes From Cox Proportional Hazards Models^∗^

## DISCUSSION

The main findings of the present study is the positive association for average SBP and J-curve association for average DBP during follow-up and risk of all-cause and CVD mortality, incident CHD, and stroke in hypertensive patients aged ≥60 years in rural areas of China. Additionally, this study demonstrated that the group with the BP of 140–149/<90 mmHg was associated with an increased risk of all-cause and CVD mortality, incident CHD, and stroke compared with the group with BP <140/90 mmHg.

In 2014, the JNC-8 panel concluded that the BP threshold and target for treatment in patient ≥60 years of age should be 150/90 mmHg rather than 140/90 mmHg, based on the RCT data available at that time.^9,20–22^ They considered this recommendation to be strongly evidence-based. However, the recommendation for less aggressive treatment in older adults has proved to be the most contentious aspect of these recommendations, even among the panel members themselves, some of whom strongly criticized their own paper in a subsequent publication.

In the post-hoc analysis from the International Verapamil SR Trandolapril Study,^13^ it was observed among hypertensive patients ≥60 years of age with CHD and baseline SBP ≥150 mmHg, that those who achieved an SBP of 140 to 150 mmHg had an increased multivariable-adjusted risk of CVD mortality, all stroke, and nonfatal stroke compared with the control group who achieved SBP <140 mmHg. Data from the Reasons for Geographic and Racial Differences in Stroke study^15^ demonstrated that patients with SBP <140 mm Hg and aged between 55 and 64 years were associated with lower incidence of all-cause mortality, CVD, CHD, and stroke, with the numerically highest incidence risk observed at SBP 140 to 149 mmHg, particularly for those ≥150 mmHg. SBP values of 130 to 139 mmHg were characterized by the numerically lowest risk of CVD incidence.^15^ Consistent with these results, our study also indicated that the older hypertensive patients with BP of 140–149/<90 mmHg had an increased risk of all-cause mortality, CVD mortality, incident CHD, and stroke compared with the reference group of BP < 140/90 mmHg. A prudent interpretation of these data suggests that hypertensive patients ≥60 years of age with BP of 140 to 149 <90 mmHg still have an increased risk of developing adverse outcomes in rural areas of China.

In addition, there is a continuous and consistent correlation between BP and risk of cardiovascular events and the notion that “lower is better” remains popular for the management of hypertension. However, this theory has been challenged for several decades, especially for DBP.^23–25^ Bangalore et al^26^ indicates that a J- or U-shaped curve association exists between BP and the risk of future cardiovascular events in patients after acute coronary syndrome, which implies that too low of a pressure (especially <110/70 mmHg) may not be an ideal target. Another post-hoc analysis of the Atrial Fibrillation Follow-up Investigation of Rhythm Management trial demonstrates a U-shaped association between average BP (SBP and DBP) and all-cause mortality in patients with atrial fibrillation (AF).^27^ The AF patients with average SBP <110 mmHg and DBP <60 mmHg had a significantly increased risk of mortality.^27^ Our analysis also revealed a J-shaped association between DBP and all-cause mortality, CVD mortality, and incident CHD in hypertensive patients ≥60 years of age. The patients with lower DBP (<85 mmHg) had an increased risk of developing adverse outcomes compared with average DBP in the range of 85 to 89 mmHg. The strict DBP control has not been shown to be superior to the lenient DBP control in older hypertensive patients. A possible explanation for the association between adverse outcomes and low DBP may be related to the high pulse pressure in our study. Since the J-shaped curve may represent an epiphenomenon of increased arterial stiffness, a low DBP might be a marker for high pulse pressure, and thus signify an increased risk of mortality and other adverse outcomes.^28^

Some limitations should also be considered in light of these results. First, the present analysis was derived from an observational epidemiological study. The results should be further validated by RCT. Second, we were lacking sufficient information on con-concomitant medications (eg, antiplatelet medications), laboratory measurements (eg, cholesterol, serum glucose, and inflammatory biomarkers), and some concomitant diseases (renal status, thyroid status, and anemia status) to control for these covariates. Third, 13.2% (n = 6080) subjects of the original cohort was lost to follow-up, which may potentially induce a degree of selection bias into the present study. These limitations, therefore, make it necessary for the results presented in this study to be regarded prudently. Finally, our study sample only included hypertensive patients aged ≥60 years in rural areas of China, and we could expect varying results from more ethnically diverse populations. The strength of our study include the relatively large sample size and large number of adverse events accrued, thereby increasing the statistical power of our analyses. Furthermore, in this study, the information on BP is derived from the average BP during follow-up, which helps us to precisely determine the association between BP and adverse outcomes.

In conclusion, in hypertensive patients aged ≥60 years of age, there was a J-shaped association between DBP and mortality and incident CHD, which suggests that too low of a DBP may not be appropriate in rural areas of China. In addition, the older hypertensive patients with BP of 140–149/<90 mmHg had a higher risk of developing adverse outcomes. These results imply that lenient BP control of 140–149/<90 mmHg, based on the JNC-8 guidelines, may not be appropriate in hypertensive patients aged ≥60 years in rural areas of China.

### Perspectives

Recent hypertension guidelines have recommended less rigid target for BP control in elderly persons. Our study demonstrates that hypertensive patients aged ≥60 years of age with BP of 140–149/<90 mmHg are at increased risk of developing adverse outcomes in rural areas of China. These present results imply that the lenient BP control of 140–149/<90 mmHg in hypertensive patients aged ≥60 years in rural areas of China may not be an ideal target. Currently, there is high prevalence of hypertension in rural China, and CVD is a major cause of death in China. Therefore, we recommend that older hypertensive patients in rural areas of China should have a therapeutic target of <140/90 mmHg. This hypothesis should be further tested in RCTs and validated by more ethnically diverse population.

## References

[1] He J Gu D Wu X Major causes of death among men and women in China. *N Engl J Med* 2005; 353:1124–1134. 16162883 10.1056/NEJMsa050467

[2] Banach M Aronow WS Hypertension therapy in the elderly adults-do we know the answers to all the questions? The status after publication of the ACCF/AHA 2011 Expert Consensus Document on Hypertension in the Elderly. *J Hum Hypertens* 2012; 26:641–643. 22513754 10.1038/jhh.2012.3

[3] Beckett NS Peters R Fletcher AE HYVET Study Group. Treatment of hypertension in patients 80 years of age or older. *N Engl J Med* 2008; 358:1887–1898. 18378519 10.1056/NEJMoa0801369

[4] Staessen JA Fagard R Thijs L Randomised double-blind comparison of placebo and active treatment for older patients with isolated systolic hypertension. The Systolic Hypertension in Europe (Syst-Eur) Trial Investigators. *Lancet* 1997; 350:757–764. 9297994 10.1016/s0140-6736(97)05381-6

[5] Fleg JL Aronow WS Frishman WH Cardiovascular drug therapy in the elderly: benefits and challenges. *Nat Rev Cardiol* 2011; 8:13–28. 20978470 10.1038/nrcardio.2010.162

[6] James PA Oparil S Carter BL 2014 Evidence-based guideline for the management of high blood pressure in adults: Report from the panel members appointed to the Eighth Joint National Committee (JNC 8). *JAMA* 2014; 311:507–520. 24352797 10.1001/jama.2013.284427

[7] Wright JT Jr Fine LJ Lackland DT Evidence supporting a systolic blood pressure goal of less than 150 mmHg in patients aged 60 years or older: the minority view. *Ann Intern Med* 2014; 160:499–503. 24424788 10.7326/M13-2981

[8] Mancia G Fagard R Narkiewicz K 2013 ESH/ESC Guidelines for the management of arterial hypertension: the Task Force for the management of arterial hypertension of the European Society of Hypertension (ESH) and of the European Society of Cardiology (ESC). *J Hypertens* 2013; 31:1281–1357. 23817082 10.1097/01.hjh.0000431740.32696.cc

[9] JATOS Study Group. Principal results of the Japanese trial to assess optimal systolic blood pressure in elderly hypertensive patients (JATOS). *Hypertens Res* 2008; 31:2115–2127. 19139601 10.1291/hypres.31.2115

[10] Ogihara T Saruta T Rakugi H Valsartan in Elderly Isolated Systolic Hypertension Study Group. Target blood pressure for treatment of isolated systolic hypertension in the elderly: Valsartan in Elderly Isolated Systolic Hypertension Study. *Hypertension* 2010; 56:196–202. 20530299 10.1161/HYPERTENSIONAHA.109.146035

[11] Zhang Y Zhang X Liu L FEVER Study Group. Is a systolic blood pressure target <140 mmHg indicated in all hypertensives? Subgroup analyses of findings from the randomized FEVER trial. *Eur Heart J* 2011; 32:1500–1508. 21345850 10.1093/eurheartj/ehr039

[12] Banach M Michalska M Kjeldsen SE What should be the optimal levels of blood pressure: does the J-curve phenomenon really exist? *Expert Opin Pharmacother* 2011; 12:1835–1844. 21517698 10.1517/14656566.2011.579106

[13] Bangalore S Gong Y Cooper-DeHoff RM 2014 Eighth Joint National Committee panel recommendation for blood pressure targets revisited: results from the INVEST study. *J Am Coll Cardiol* 2014; 64:784–793. 25145522 10.1016/j.jacc.2014.05.044 PMC4193384

[14] Gradman AH Optimal blood pressure targets in older adults: how low is low enough? *J Am Coll Cardiol* 2014; 64:794–796. 25145523 10.1016/j.jacc.2014.06.1153

[15] Banach M Bromfield S Howard G Association of systolic blood pressure levels with cardiovascular events and all-cause mortality among older adults taking antihypertensive medication. *Int J Cardiol* 2014; 176:219–226. 25085381 10.1016/j.ijcard.2014.07.067 PMC4144437

[16] Aronow WS Fleg JL Pepine CJ ACCF/AHA 2011 Expert consensus document on hypertension in the elderly: a report of the American College of Cardiology Foundation Task Force on clinical expert consensus documents. *Circulation* 2011; 123:2434–2506. 21518977 10.1161/CIR.0b013e31821daaf6

[17] Fleg JL Aronow WS Frishman WH Cardiovascular drug therapy in the elderly: benefits and challenges. *Nat Rev Cardiol* 2011; 8:13–28. 20978470 10.1038/nrcardio.2010.162

[18] Zheng L Sun Z Zhang X Predictive value for the rural Chinese population of the Framingham hypertension risk model: results from Liaoning Province. *Am J Hypertens* 2014; 27:409–414. 24308978 10.1093/ajh/hpt229

[19] Harrell FE Jr Lee KL Mark DB Multivariable prognostic models: issues in developing models, evaluating assumptions and adequacy, and measuring and reducing errors. *Stat Med* 1996; 15:361–387. 8668867 10.1002/(SICI)1097-0258(19960229)15:4<361::AID-SIM168>3.0.CO;2-4

[20] Cushman WC Evans GW ACCORD Study Group. Effects of intensive blood-pressure control in type 2 diabetes mellitus. *N Engl J Med* 2010; 362:1575–1585. 20228401 10.1056/NEJMoa1001286 PMC4123215

[21] Pepine CJ Handberg EM Cooper-DeHoff RM A calcium antagonist vs a non-calcium antagonist hypertension treatment strategy for patients with coronary artery disease. The International Verapamil-Trandolapril Study (INVEST): a randomized controlled trial. *JAMA* 2003; 290:2805–2816. 14657064 10.1001/jama.290.21.2805

[22] Pepine CJ Handberg-Thurmond E Marks RG Rationale and design of the International Verapamil SR/Trandolapril Study (INVEST): an Internet-based randomized trial in coronary artery disease patients with hypertension. *J Am Coll Cardiol* 1998; 32:1228–1237. 9809930 10.1016/s0735-1097(98)00423-9

[23] Cruickshank JM Thorp JM Zacharias FJ Benefits and potential harm of lowering high blood pressure. *Lancet* 1987; 1:581–584. 2881129 10.1016/s0140-6736(87)90231-5

[24] Lindholm L Lanke J Bengtsson B Both high and low blood pressures risk indicators of death in middle-aged males: isotonic regression of blood pressure on age applied to data from a 13-year prospective study. *Acta Med Scand* 1985; 218:473–480. 4091047 10.1111/j.0954-6820.1985.tb08876.x

[25] Messerli FH Mancia G Conti CR Dogma disputed: can aggressively lowering blood pressure in hypertensive patients with coronary artery disease be dangerous? *Ann Intern Med* 2006; 144:884–893. 16785477 10.7326/0003-4819-144-12-200606200-00005

[26] Bangalore S Qin J Sloan S PROVE IT-TIMI 22 Trial Investigators. What is the optimal blood pressure in patients after acute coronary syndromes? Relationship of blood pressure and cardiovascular events in the PRavastatin OR atorVastatin Evaluation and Infection Therapy-Thrombolysis in Myocardial Infarction (PROVE IT-TIMI) 22 trial. *Circulation* 2010; 122:2142–2151. 21060068 10.1161/CIRCULATIONAHA.109.905687

[27] Badheka AO Patel NJ Grover PM Optimal blood pressure in patients with atrial fibrillation (from the AFFIRM Trial). *Am J Cardiol* 2014; 114:727–736. 25060415 10.1016/j.amjcard.2014.06.002

[28] Kannel WB Wilson PW Nam BH A likely explanation for the J-curve of blood pressure cardiovascular risk. *Am J Cardiol* 2004; 94:380–384. 15276113 10.1016/j.amjcard.2004.04.043

